# Track-to-Track Fusion for Cooperative Perception Using Collective Perception Messages

**DOI:** 10.3390/s26062003

**Published:** 2026-03-23

**Authors:** Redge Melroy Castelino, Shrijal Pradhan, Axel Hahn

**Affiliations:** 1 Institute of Systems Engineering for Future Mobility, German Aerospace Center (DLR), 26121 Oldenburg, Germany; shrijal.pradhan@dlr.de (S.P.); axel.hahn@dlr.de (A.H.); 2 Department of Computing Science, Carl von Ossietzky University, 26129 Oldenburg, Germany

**Keywords:** cooperative perception, collective perception messages, high-level data fusion, track-to-track fusion, vehicle-to-everything

## Abstract

Vehicle-to-everything communication grants connected and automated road vehicles the opportunity to share their sensor information such as detected road objects for collective awareness. This paper compares various state fusion strategies within a high-level cooperative perception architecture, focusing on the fusion of object-level information provided in standard Collective Perception Messages. This work compares five track-to-track fusion methods, namely Covariance Intersection, Inverse Covariance Intersection, Adapted Extended Kalman Filter, Adapted Unscented Kalman Filter and Information Matrix Fusion, using a simulation framework built with CARLA and Autoware. The methods are analyzed in a case study to assess their performance under different vehicle maneuvers and varying input information accuracy. The case study highlights trade-offs between fusion strategies and illustrate their behavior in asynchronous multi-agent scenarios. While the analysis is conducted in simulation, the architecture is designed to be extensible, and directions for future development are outlined, including the integration of classification and object confidence fusion modules.

## 1. Introduction

The development of cooperative perception systems has emerged as a key strategy to enhance the perception capabilities of connected and automated vehicles (CAVs), especially in complex urban environments where local sensing is often constrained by occlusions and limited sensor range [1,2,3,4,5]. A central enabler of this paradigm is the exchange of perception information through vehicle-to-everything (V2X) communication, specifically via Collective Perception Messages (CPMs). CPMs are designed to transmit abstracted representations of perceived objects, rather than raw sensor data, thereby enabling interoperability across heterogeneous Intelligent Transportation System Stations (ITS-S), including connected vehicles and intelligent roadside units. CPMs contain multiple structured containers that include object-level descriptions, sensor metadata, and pose information to facilitate efficient communication of perception data across diverse ITS-S. Despite growing standardization in the structure and content of CPMs, the fusion of received data into a unified and interpretable model remains a persistent research problem [6]. This work analyzes various track-to-track fusion methods for V2X-based cooperative perception, specifically targeting the integration of object-level data received through CPMs. Section 2 provides an overview of related works of fusion methods for cooperative perception in intelligent transport systems. Building on the data models and architectural considerations specified in [7,8], we present a high-level fusion architecture design to enable scalable, robust, and consistent integration of CPM-based perception across multiple ITS-S in Section 3. Section 4 details and summarizes state-of-the-art track-to-track fusion algorithms, which are analyzed in a case study elaborated in Section 5. Finally, Section 6 provides a discussion of the findings and outlines limitations of the experimental setup and future work.

## 2. Related Works

In recent years, several studies have investigated the fusion of ITS-S perception data for cooperative road-object detection and tracking. Cooperative fusion strategies are typically categorized into three levels: low-level or early fusion, which involves combining raw sensor data; feature-level or intermediate fusion, where intermediate features or processing results are shared and fused; and high-level or late fusion, which focuses on the integration of processed vehicle perception outputs such as object-level information or tracks.

In low-level or early fusion, each transmitting ITS-S sends raw sensor data through the communication medium. The receiving ITS-S transforms the received data into a common coordinate frame prior to further processing, thereby enabling an expansion of the sensor data range and/or density [9,10]. However, performance of early fusion approaches are sensitive to sensor calibration issues and out-of-sequence measurements. Furthermore, early fusion approaches require large communication bandwidth to transmit high volumes of raw data [10,11,12].

Intermediate or deep fusion exchanges learned feature maps or latent queries instead of raw data or final detections. This can preserve most task-relevant information while substantially reducing communication volume and improving robustness to localization errors and communication delays compared to early fusion. However, intermediate fusion still faces limitations. Exchanging high-dimensional feature maps can remain bandwidth-intensive when many agents participate, especially in dense traffic scenarios. In addition, the real-time behavior of increasingly complex fusion backbones, such as transformer-based architectures, is not yet well established under realistic V2X latency, jitter, and packet loss conditions. Finally, current methods still struggle with robustness to pose errors, heterogeneous ITS-S perception architectures, and distribution shifts between agents and environments, especially as the number of ITS-S grow [12,13,14].

High-level fusion (also called late fusion) combines object-level outputs from multiple ITS-S, such as tracks, bounding boxes, and class labels, into a unified scene representation instead of sharing raw data or intermediate features. This fusion is typically performed on bounding boxes, kinematic states, and class labels, and they can therefore leverage heterogeneous local perception stacks while keeping the communication payload compact, which is favored in bandwidth-constrained V2X networks [9,15].

In recent high-level data fusion for cooperative perception have been demonstrated in several real-world use-cases [15,16,17,18,19] due to several key advantages:

- Communication Efficiency: High-level fusion transmits only essential object-level information, significantly reducing bandwidth usage compared to early or intermediate fusion, which would require sending raw sensor data or large feature maps containing data volumes that current V2X technologies cannot reliably support [20,21,22].
- Robustness and Scalability: By operating on processed object-level data, high-level fusion is more resilient to communication disruptions and packet loss. It also enables straightforward integration across heterogeneous platforms, as object-level information is standardized and less dependent on specific sensor modalities [20,21,22].
- Improved Perception Quality: Fusing object-level data from multiple sources allows for the resolution of ambiguities, filtering of false positives, and overall improvement in detection accuracy, especially in complex or occluded environments [21].
- Alignment with Industry CPM standards: In recent years, V2X services such as the Collective Perception Service have been standardized, which includes the CPM message format data elements and functional specification [7,8]. CPMs transmit perceived object-level data, making high-level fusion a natural and efficient fit for the information exchanged in current and future V2X cooperative perception systems [20,21].

High-level fusion frameworks for cooperative perception typically comprise two fusion steps. The first involves fusion of data arriving from local perception sensors available to the ITS-S in order to generate its local environment model. In the next step, object-level information from the local environment model are fused with arriving V2X messages, such as the CPM, to generate a global or V2X-enriched environment model [15,18]. Object-level data from both stages are available downstream for situation assessment and planning. Several works describe track-to-track fusion methods for high-level or late fusion of object data for cooperative perception. Ref. [16] investigates covariance intersection (CI) fusion to handle possible track cross-correlation and demonstrates reduction in estimation uncertainty after global fusion. Ref. [19] presents a test field architecture, where object-level data is communicated via V2X from multiple roadside units and a CAV, and fused using an Unscented Kalman Filter (UKF) for a traffic monitoring use case. Similarly, ref. [15] presents experimental results of an UKF in a high-level fusion architecture when fusing CPMs from multiple CAVs.

While several studies have demonstrated the feasibility of high-level cooperative perception using object-level data exchanged via V2X communication, existing works typically focus on individual fusion approaches or application-specific architectures. A comparison of multiple track-to-track fusion algorithms under identical experimental conditions while explicitly considering the constraints imposed by the Collective Perception Message (CPM) specification [7,8], such as data structure, and CPM generation rules remains limited in the literature. To address this gap, this work investigates and compares several representative track-to-track fusion strategies within a CPM-compliant cooperative perception framework. The algorithms are implemented in a controlled CARLA–Autoware simulation environment and compared in a case study involving varying vehicle maneuvers and input data quality. The presented case study, described in Section 5, provides a case study analysis of the behavior, accuracy, and statistical consistency of these fusion methods when applied to CPM-based cooperative perception.

## 3. High-Level Fusion Architecture for Collective Perception Messages

**Figure 1:**
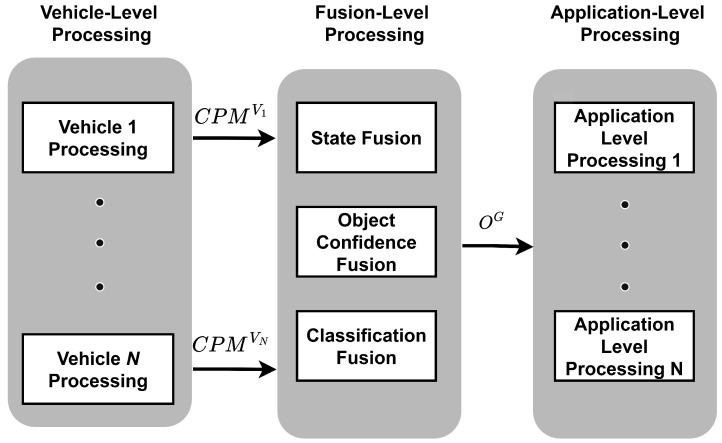
High-level fusion architecture for collective perception. Adapted from ref. [23].

The proposed high-level fusion architecture for fusion of V2X messages, adapted from ref. [23], integrates V2X CPMs to build a robust environment model. This architecture is divided into three main processing levels: Vehicle-Level Processing, Fusion-Level Processing, and Application-Level Processing. Figure 1 illustrates the overall high-level fusion architecture.

### 3.1. Vehicle-Level Processing

At the vehicle level, sensor data from a CAV’s local perception stack is processed and fused into an environment model representing the vehicle’s surroundings. The perceived road objects are then abstracted into the standard CPM format and broadcast via V2X communication. This standardized data model ensures modularity and interoperability, allowing integration across diverse hardware and software platforms.

### 3.2. Fusion-Level Processing

The core of the architecture lies in fusion-level processing, where CPMs from different vehicles are received and integrated into a unified global environment model. The output of this process is a global object list $O^{G}$, in which each object is represented by a state vector ${\hat{x}}^{G}$, covariance matrix $P^{G}$, object confidence or existence probability $p{\left(\exists x\right)}^{G}$, and classification vector $c^{G}$:

(1) $$O^{G}=\left\{{\hat{x}}^{G},P^{G},p{\left(\exists x\right)}^{G},c^{G}\right\}$$

The state vector ${\hat{x}}^{G}$ is described by the longitudinal and lateral positions $p_{x}$ and $p_{y}$, absolute velocity *v*, heading angle θ, and yaw rate ψ of the perceived object.

(2) $${\hat{x}}^{G}={\left[p_{x}^{G},p_{y}^{G},v^{G},\theta^{G},\psi^{G}\right]}^{T}$$

The superscript in the above described variables represents the track to which the variable belongs. *G* represents the global fused track and $V_{i}$ represents the track belonging to vehicle *i*.

**Figure 2:**
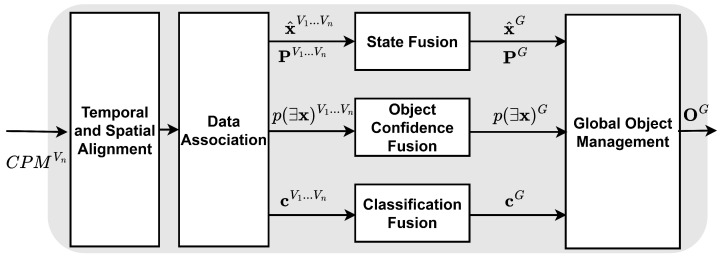
Fusion-level processing structure for producing global object list. Adapted from [23].

The goal at the fusion level is to produce stable and robust global tracks of objects that may appear in the sensor fields of view of multiple vehicles. The system must handle asynchronous and out-of-sequence measurements. Figure 2 illustrates the fusion-level processing structure.

#### 3.2.1. Spatial Alignment

For spatial alignment of vehicle-level tracks into the global reference frame, rotational transformations are represented using unit quaternions rather than Euler angles or rotation matrices. Quaternion-based representations avoid singularities such as gimbal lock and provide improved numerical stability when composing rotations, which is a consideration for spatial alignment of perception information arriving from infrastructure [24]. A rotation is represented by a unit quaternion:

(3) $$q={\left[\begin{array}{cccc} q_{w} & q_{x} & q_{y} & q_{z} \end{array}\right]}^{⊤}$$

where $q_{w}$ denotes the scalar component and ${\left[q_{x},q_{y},q_{z}\right]}^{⊤}$ represents the vector part. The quaternion is constrained to unit norm, ∥q∥=1.

A perceived object position $p_{V}\in R^{3}$ expressed in a local vehicle coordinate frame is transformed into the global frame as

(4) $$p_{G}=q⊗p_{V}⊗q^{-1}+t$$

where ⊗ denotes quaternion multiplication, $q^{-1}$ is the quaternion inverse, and t represents the translation column vector between the local and global frames.

The rotation quaternion *q* is constructed from the yaw (ψ), pitch (θ), and roll (ϕ) angles provided in the CPM’s originating station data container, following specifications in [7]. These Euler angles, expressed relative to the WGS84 coordinate frame [25], are converted to a unit quaternion using the standard yaw–pitch–roll sequence.

The translation vector t is obtained directly from the reference position (longitude, latitude, altitude) in the CPM’s originating station data, converted to local Cartesian coordinates. This ensures proper spatial alignment of object-level perception data into the global fusion frame. The same transformation is applied to the positional components of the vehicle-level state vector prior to track-to-track fusion.

#### 3.2.2. Fusion Strategy and Temporal Alignment

**Figure 3:**
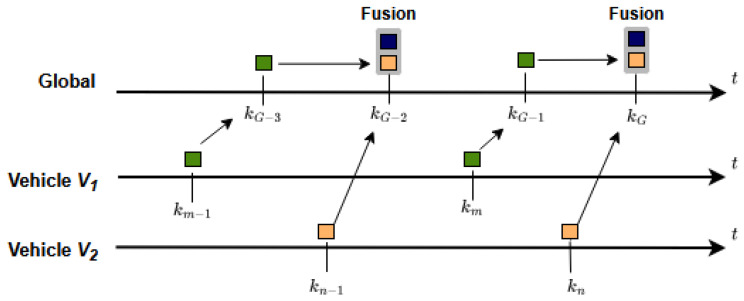
Peer-to-peer fusion strategy.

**Figure 4:**
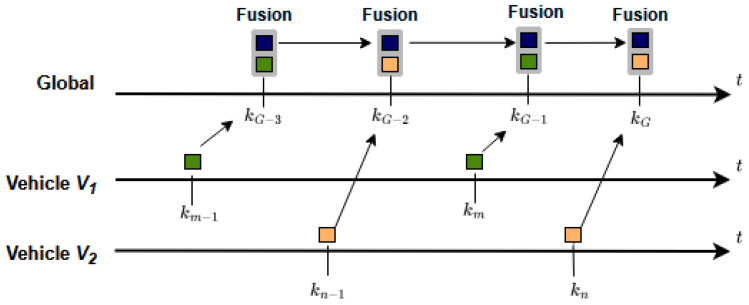
Peer-to-global fusion strategy.

There are two possible strategies that could be employed for temporal alignment in track-to-track fusion, which are illustrated in Figure 3 and Figure 4.

Figure 3 illustrates a peer-to-peer strategy (also known as sensor-to-sensor fusion), where local tracks from multiple vehicles are predicted to a predefined fusion cycle time and then merged to create a global track. Since this method ignores previously fused global information in subsequent cycles, it is often described as track-to-track fusion with no memory [26].

In contrast, the peer-to-global strategy (sensor-to-global fusion) maintains a persistent global track that is updated incrementally as new input tracks arrive [27]. Figure 4 illustrates a peer-to-global strategy, where vehicle level tracks $V_{1}$ and $V_{2}$ are asynchronously predicted and fused to the global track. This memory-based approach is highly effective when objects transition between the fields of view of different vehicles, ensuring continuity and stability in object tracking. Owing to these advantages, the current work focuses exclusively on peer-to-global state fusion. Since the a priori state of the global track is fused with the asynchronous inputs upon arrival, it is often also called track-to-track fusion with memory [26].

In our work, we adopt a peer-to-global strategy using a constant velocity and turn rate (CVTR) model to predict tracks for appropriate temporal alignment of asynchronously arriving CPMs.

#### 3.2.3. Data Association

After spatial and temporal alignment, it is essential to identify which perceived objects in the received CPM should be fused with objects from the last fusion step in the global track. Incorrect data association while fusing CPMs can result in ghost objects (false positives), missed detection (false negatives), ID switches, and degradation of the global or fused track accuracy and stability [28]. We adopt a Mahalanobis distance-based object data association to match perceived objects in the incoming CPMs with the corresponding entries in the global track database. We implement the Jonker-Volgenant algorithm-based association [29], followed by fusion of associated objects.

#### 3.2.4. Fusion

After object association, the state and covariance, existence, and classification for the associated objects from their respective tracks are fused at three levels:

- State Fusion: The reconstructed state vector ${\hat{x}}^{G}$ and covariance matrix $P^{G}$ from the input CPM are fused with track-to-track fusion approaches with a peer-to-global strategy. We further detail some track-to-track fusion algorithms in Section 4.
- Object Confidence Fusion: Perceived objects in the CPM are provided with object confidence scores describing the confidence of the sensing ITS station. Depending on factors like distance from the object, detecting sensor characteristics, and road situation, different transmitting ITS-S would have different confidence scores for the same detected object. Object confidence fusion aggregates confidence scores or existence probabilities for every perceived object across multiple CPMs into a global object existence score. For example, ref. [30] introduces the use of Dempster–Shafer Theory (DST) for the purpose of aggregating object existence probabilities from multiple sensor-level detections.
- Classification Fusion: Aggregates classification probabilities or scores for perceived objects from CPMs. Several approaches are applicable here, including the Multi-modal Multi-class Late Fusion approach [31] and DST-based class fusion [32].

While the proposed architecture includes modules for state, existence, and classification fusion, the experimental evaluation presented in this work focuses on the state fusion component. This choice enables a controlled comparison of different track-to-track fusion strategies while keeping the remaining perception pipeline unchanged. Incorporating existence and classification fusion introduces additional modeling assumptions related to object confidence aggregation and semantic consistency across agents, which are beyond the scope of the present study. These components are therefore treated as architectural extensions and will be addressed in future work.

### 3.3. Application-Level Processing

At the application level, the global objects are further processed and filtered for specific applications such as Advanced Driver Assistance Systems (ADAS) [33] and traffic monitoring applications [34,35].

## 4. Track-to-Track Fusion Algorithms

In this section, we discuss track-to-track fusion algorithms for a peer-to-global strategy for the state fusion of detected objects from incoming CPMs. This type of strategy is suitable for asynchronous, distributed, and multi-sensor environments in which CAVs operate, as will be demonstrated in Section 5.

We adopt the following convention to describe the state vector ${\hat{x}}^{G}\left(k_{G}|k_{m}\right)$ and covariance matrix $P(k_{G}|k_{m})$, where the first index denotes the time step of the state, and the second index denotes the time step of the information (conditioning data) used to compute the estimate. Specifically, $x^{G}\left(k_{G}|k_{G-1}\right)$ represents the predicted (a priori) global state at time $k_{G}$, obtained using all measurements available up to time $k_{G-1}$. This estimate is typically produced by propagating the system model forward in time without incorporating the current measurement.

In contrast, ${\hat{x}}^{G}\left(k_{G}|k_{G}\right)$ denotes the updated (a posteriori) global state at the same time instant $k_{G}$, after incorporating the measurement available at time $k_{G}$. This estimate reflects both the system dynamics and the information provided by the current observation.

The proposed formulation relies on several standard assumptions commonly adopted in automotive multi-object tracking and V2X data fusion:

- Vehicle-level track estimates derived from CPMs are assumed to be represented by Gaussian state distributions with associated covariance information, enabling Kalman Filter-based fusion.
- CPM messages are assumed to provide reliable timestamps that allow incoming track estimates to be temporally aligned with the global fusion timeline.
- Apart from correlations introduced by shared process histories, vehicle-level tracks are treated as conditionally independent.

These assumptions are consistent with current CPM specifications and state-of-the-art perception pipelines; relaxing them to account for non-Gaussian uncertainties or imperfect time synchronization is left for future work.

In the following subsections, we present and summarize a selection of track-to-track fusion algorithms suitable for a peer-to-global strategy in asynchronous V2X environments. The present approachs originate from automotive multi-sensor fusion [15,36,37,38,39,40,41]. In the following subsections we use Figure 4 as a reference, referring specifically to the last update from vehicle-level track $V_{2}$ to the global track *G*.

### 4.1. Covariance Intersection Fusion

CI is a fusion approach that combines two or more estimates when the correlation among them is unknown. This approach takes the convex combination of mean and covariance estimates in the information (or inverse covariance) space [36]. The fused covariance is calculated as follows:

(5) $$P^{G}\left(k_{G}|k_{G}\right)={\left[\omega P^{G}{\left(k_{G}|k_{G-1}\right)}^{-1}+\left(1-\omega \right)P^{V_{2}}{\left(k_{G}|k_{n}\right)}^{-1}\right]}^{-1}$$

where $P^{G}\left(k_{G}|k_{G-1}\right)$ is the covariance of the previously fused global track predicted to the current time, and $P^{V_{2}}\left(k_{G}|k_{m}\right)$ is the covariance of the input from vehicle track $V_{2}$, predicted to the arrival time at the fusion module.

The fused state estimate is calculated as

(6) $$\begin{array}{c} {\hat{x}}^{G}\left(k_{G}|k_{G}\right)=P^{G}{\left(k_{G}|k_{G}\right)}^{-1}[\omega P^{G}{\left(k_{G}|k_{G-1}\right)}^{-1}{\hat{x}}^{G}\left(k_{G}|k_{G-1}\right)\\ \;+\left(1-\omega \right)P^{V_{2}}{\left(k_{G}|k_{n}\right)}^{-1}{\hat{x}}^{V_{2}}\left(k_{G}|k_{n}\right)] \end{array}$$

where ${\hat{x}}^{G}\left(k_{G}|k_{G-1}\right)$ is the state estimate from the previously fused global track, and ${\hat{x}}^{V_{2}}\left(k_{G}|k_{n}\right)$ is the state estimate of input from the vehicle-level track, both predicted to the arrival time of the input at the global track.

The weighing factor ω is obtained by optimization of the fused covariance to minimize the determinant

(7) $$\omega =argmin(det\left(P^{G}\left(k_{G}|k_{G}\right)\right)).$$

CI considers the correlation between the multiple state estimates and attempts to fuse them while maintaining consistency. It has been popularly used in both peer-to-peer and peer-to-global data fusion strategies for automotive sensor data fusion applications [37,38,39] as well as in V2X data fusion applications [16].

### 4.2. Inverse Covariance Intersection Fusion

The inverse covariance intersection (ICI) is a decentralized data fusion approach derived in [42] to address the challenge of state estimates that share unknown common information. ICI yields less conservative covariance bounds than CI by estimating maximum feasible common information through inverse covariance intersection, while guaranteeing estimator consistency. The approach has demonstrated consistent results in fusion problems involving estimates with common process noise, making it suitable for peer-to-global fusion strategies [43]. The fused state estimate and covariance are calculated as follows:

(8) $${\hat{x}}^{G}\left(k_{G}|k_{G}\right)=K_{ICI}{\hat{x}}^{G}\left(k_{G}|k_{G-1}\right)+L_{ICI}{\hat{x}}^{V_{2}}\left(k_{G}|k_{n}\right)$$

(9) $$\begin{array}{c} P^{G}\left(k_{G}|k_{G}\right)=[P^{G}{\left(k_{G}|k_{G-1}\right)}^{-1}+P^{V_{2}}{\left(k_{G}|k_{n}\right)}^{-1}-\\ \;{\left(\omega P^{G}\left(k_{G}|k_{G-1}\right)+\left(1-\omega \right)P^{V_{2}}\left(k_{G}|k_{n}\right)\right)}^{-1}{]}^{-1} \end{array}$$

where fusion gains $K_{ICI}$ and $L_{ICI}$ are given by

(10) $$K_{ICI}=P^{G}\left(k_{G}|k_{G}\right)\left[P^{G}{\left(k_{G}|k_{G-1}\right)}^{-1}-\omega {\left(\omega P^{G}\left(k_{G}|k_{G-1}\right)+\left(1-\omega \right)P^{V_{2}}\left(k_{G}|k_{n}\right)\right)}^{-1}\right]$$

(11) $$L_{ICI}=P^{G}\left(k_{G}|k_{G}\right)\left[P^{V_{2}}{\left(k_{G}|k_{n}\right)}^{-1}-\left(1-\omega \right){\left(\omega P^{G}\left(k_{G}|k_{G-1}\right)+\left(1-\omega \right)P^{V_{2}}\left(k_{G}|k_{n}\right)\right)}^{-1}\right]$$

The weighing factor ω is determined by optimization of the fused covariance to minimize its determinant as described in Equation (7).

### 4.3. Adapted Kalman Filter

One approach for a peer-to-global fusion strategy is to use peer-level track as measurement inputs to a global Kalman Filter (KF) algorithm.

The following equations describe the equations for a KF adapted to update vehicle-level tracks from vehicle $V_{2}$ at time $k_{n}$ to the global track at $k_{G}$. For a peer-to-global track fusion strategy, the state and covariance of the global track must be predicted to the measurement arrival time $k_{G}$ of the vehicle-level track $V_{2}$.

(12) $${\hat{x}}^{G}\left(k_{G}|k_{G-1}\right)=F\left(k_{G},k_{G-1}\right){\hat{x}}^{G}\left(k_{G-1}|k_{G-1}\right)$$

(13) $$P^{G}\left(k_{G}|k_{G-1}\right)=F\left(k_{G},k_{G-1}\right)P^{G}\left(k_{G-1}|k_{G-1}\right)F{\left(k_{G},k_{G-1}\right)}^{T}+Q\left(k_{G},k_{G-1}\right)$$

where $F(k_{G},k_{G-1})$ is the linear state transition function (e.g.,- constant velocity model) and $Q(k_{G},k_{G-1})$ is the process noise.

(14) $$S\left(k_{G}\right)=P^{G}\left(k_{G}|k_{G-1}\right)+P^{V_{2}}\left(k_{G}|k_{n}\right)$$

(15) $$K\left(k_{G}\right)=P^{G}\left(k_{G}|k_{G-1}\right)S{\left(k_{G}\right)}^{-1}$$

(16) $${\hat{x}}^{G}\left(k_{G}|k_{G}\right)={\hat{x}}^{G}\left(k_{G}|k_{G-1}\right)+K\left(k_{G}\right)\left[{\hat{x}}^{V_{2}}\left(k_{G}|k_{n}\right)-{\hat{x}}^{G}\left(k_{G}|k_{G-1}\right)\right]$$

(17) $$P^{G}\left(k_{G}|k_{G}\right)=\left[1-K(k_{G})\right]P^{G}\left(k_{G}|k_{G-1}\right)$$

where ${\hat{x}}^{V_{2}}\left(k_{G}|k_{n}\right)$ and $P^{V_{2}}\left(k_{G}|k_{m}\right)$ are the input state vector and covariance reconstructed from the input CPM, $S(k_{G})$ is the innovation covariance, $K(k_{G})$ is the Kalman gain, and ${\hat{x}}^{G}\left(k_{G}|k_{G}\right)$ and $P^{G}\left(k_{G}|k_{G}\right)$ are the updated global state estimate and covariance, respectively.

Since vehicle-level tracks are correlated with one another due to their common information history, the assumption that measurements to the KF are independent from one another is violated. In the adopted formulation, retrodiction is used to align asynchronous vehicle-level track updates with the internal timeline of the global track. When a vehicle-level estimate arrives with a timestamp earlier than the current global time, the global state is first retrodicted to the measurement time, fused with the incoming track estimate, and subsequently predicted forward again. This procedure prevents double counting of shared process noise and preserves estimator consistency in the presence of correlated track histories and out-of-sequence information. Since the mathematical formulation of retrodiction is well established, the reader is referred to [23,44].

The presented KF Equations (12) and (13) assume a linear state transition model and additive Gaussian process noise, which allows the use of a standard KF for prediction and update. However, vehicle motion models that explicitly capture turning behavior—such as CTRV models—are non-linear in the state variables. To accommodate such non-linear system dynamics, the prediction step in Equations (12) and (13) can be generalized using a non-linear state transition function.

(18) $${\hat{x}}^{G}\left(k_{G}|k_{G-1}\right)=f\left({\hat{x}}^{G}\left(k_{G-1}|k_{G-1}\right),u_{G-1}\right)$$

where f() is the non-linear state transition function (e.g., constant velocity constant turn-rate model).

In this case, an Extended Kalman Filter (EKF) may be employed by linearizing the non-linear transition function around the current state estimate using a first-order Taylor expansion. The resulting Jacobian matrix replaces the linear state transition matrix $F(k_{G},k_{G-1})$ in the prediction step. In this work, we refer to a global EKF, which employs retrodiction to handle correlation and out-of-sequence vehicle-level tracks, referred to as the Adapted EKF.

Alternatively, an UKF can be used to address non-linearities without explicit linearization. The UKF propagates a deterministic set of sigma points through the non-linear transition function and reconstructs the predicted mean and covariance, often yielding improved estimation accuracy for highly non-linear maneuvers. In this work, we refer to a global UKF, which employs retrodiction to handle correlation and out-of-sequence vehicle-level tracks as the Adapted UKF.

Several works demonstrate the effectiveness of using various versions of the KF for automotive sensor data fusion applications [39,45,46,47,48] as well as V2X data fusion applications [15,40,41].

### 4.4. Information Matrix Fusion

Information Matrix Fusion (IMF) is a track-to-track fusion approach, where cross-covariance between tracks is explicitly calculated. However, the algorithm is required to retain memory of previous updates from peer-level tracks in order to calculate information gain, as indicated in Equations (19) and (20), that calculate the fused covariance and state.

(19) $$P^{G}{\left(k_{G}|k_{G}\right)}^{-1}=P^{G}{\left(k_{G}|k_{G-1}\right)}^{-1}+\left[P^{V_{2}}{\left(k_{G}|k_{n}\right)}^{-1}-P^{V_{2}}{\left(k_{G}|k_{n-1}\right)}^{-1}\right]$$

(20) $$\begin{array}{c} x^{G}{\left(k_{G}|k_{G}\right)}^{-1}=P^{G}{\left(k_{G}|k_{G}\right)}^{-1}(P^{G}{\left(k_{G}|k_{G-1}\right)}^{-1}x^{G}\left(k_{G}|k_{G-1}\right)\\ \;+\left[P^{V_{2}}{\left(k_{G}|k_{n}\right)}^{-1}x^{V_{2}}\left(k_{G}|k_{n}\right)-P^{V_{2}}{\left(k_{G}|k_{n-1}\right)}^{-1}x^{V_{2}}\left(k_{G}|k_{n-1}\right)\right]) \end{array}$$

The IMF formulation requires retaining the previous vehicle-level track estimate in order to compute the information increment between consecutive updates. For the state vector used in this work $x={\left[p_{x},p_{y},v,\theta ,\psi \right]}^{T}$, the covariance matrix has dimension 5×5. Assuming double-precision floating point representation (8 bytes), storing a state vector and covariance matrix requires approximately 240 bytes. Since IMF requires maintaining the previous estimate in addition to the current estimate, the memory overhead per tracked object is approximately 480 bytes. Even for scenarios involving dozens of tracked objects and multiple contributing vehicles, the resulting memory requirement remains on the order of a few tens of kilobytes, which is negligible relative to the available memory resources of typical automotive compute platforms. IMF has been demonstrated for automotive local sensor data fusion applications with peer-to-global strategies in [39,49].

Table 1 summarizes the key strengths and limitations of the evaluated track-to-track fusion approaches in the context of peer-to-global cooperative perception using CPMs. Here, Adapted KF collectively represents the Adapted EKF and Adapted UKF presented in Section 4.3.

## 5. Case Study

### 5.1. Experiment Setup

**Figure 5:**
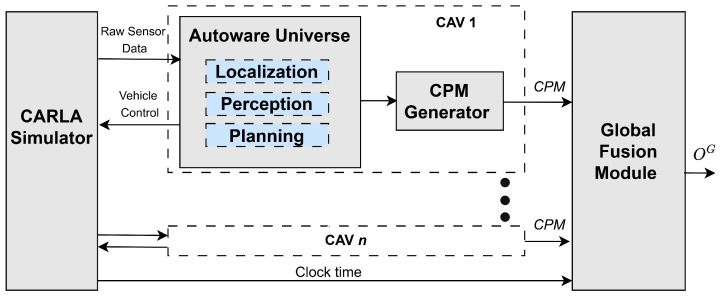
CARLA–Autoware Experiment Setup.

The fusion algorithms discussed in Section 4 are analyzed and compared using the open-source simulator CARLA [50]. We employ Autoware Universe [51], a ROS2-based open-source automated driving stack, to control CAVs within the simulation, as illustrated in Figure 5.

We further extend Autoware to generate CPMs, as per specification in [7,8]. CPMs are generated and transmitted at periodic generation events within bounded intervals, rather than being broadcast continuously. Perceived objects are included in the CPM only if specific triggering conditions are satisfied, such as the detection of a new object or when the estimated object state has changed beyond predefined thresholds (e.g., changes in position, ground speed, or velocity orientation) relative to the last reported state, or when a maximum reporting interval has elapsed. In our implementation, the confidence values for different measurements in the CPM are populated using the corresponding diagonal elements from the covariance matrix from the Autoware local tracker. The CPMs from multiple CAVs are subscribed to and fused to generate a high-confidence global environment model in the fusion module.

During the simulation run, a state vector and covariance matrix are reconstructed from the CPM for every perceived object and then fused into a global track using the presented track-to-track fusion approaches.

### 5.2. Results

**Figure 6:**
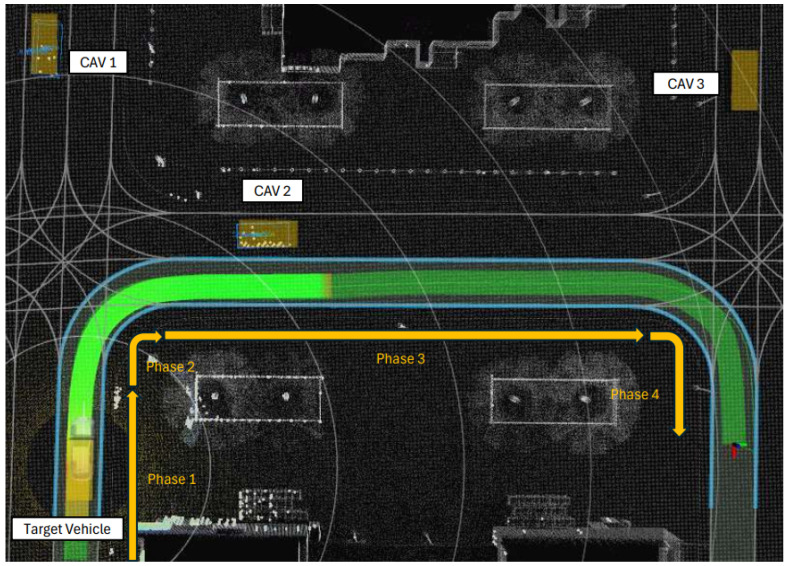
Case study scenario in CARLA Town04. Every orange box in the scenario indicates a CAV in the scenario. The green path indicates the trajectory executed by the Ego vehicle.

We perform an analysis of the presented track-to-track fusion algorithms for collective perception, considering a scenario where a target vehicle moves through the perception range of three different CAVs, as illustrated in Figure 6. The scenario can be divided into 4 phases. Phase 1 describes a straight line motion of the target vehicle entering an intersection. Phase 2 describes a turning motion of the target vehicle as it is detected by two CAVs: CAV 1 and CAV 2. Phase 3 describes straight line acceleration of the target vehicle on a straight road, and Phase 4 the turning and braking motion of the target.

Every CAV is equipped with a 360-degrees, 64-channel Lidar that is used for the object detection task. To simulate the loss due to external factors, a point drop-off rate of 0.45 is set. The simulated Lidar point cloud is processed by the Autoware software stack to detect and track road objects using a center-based 3D object detection and tracking [52]. The tracked objects are then shared as perceived objects in the transmitted CPM as illustrated in Figure 5.

The state fusion algorithms are implemented as ROS2 nodes that subscribe to CPM messages published by the individual CAVs. For every perceived object contained in an incoming CPM, a state vector and associated covariance matrix are reconstructed and temporally aligned with the global fusion timeline. The reconstructed vehicle-level track is then fused into a persistent global track using the respective peer-to-global fusion strategy asynchronously. Both the fused global estimates and the CARLA ground-truth states are recorded as ROS2 bag files and analyzed offline.

**Figure 7:**
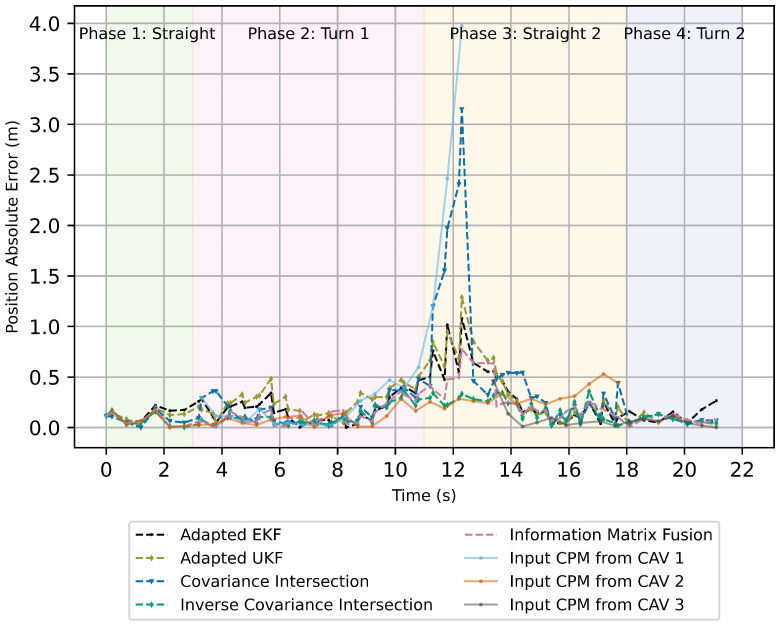
Absolute error for position estimation from track-to-track fusion algorithms using a peer-to-global fusion strategy. The solid lines indicate the absolute error in input CPMs and the dashed lines indicate the absolute error in position estimates.

**Figure 8:**
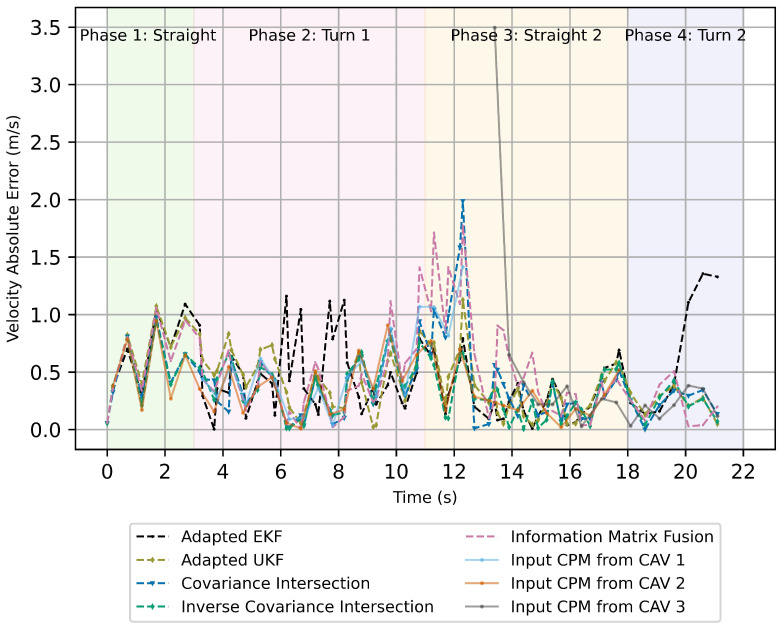
Absolute error for velocity estimation from track-to-track fusion algorithms using a peer-to-global fusion strategy. The solid lines indicate the absolute error in input CPMs and the dashed lines indicate the absolute error in fused velocity estimates.

Figure 7 and Figure 8 presents the absolute errors of position and velocity estimates for the analyzed track-to-track fusion algorithms. During Phase 1, where the target vehicle follows near-linear motion, all track-to-track fusion approaches exhibit low and comparable position and velocity errors. The CVTR model is well aligned with the true vehicle dynamics in this regime, and the asynchronous peer-to-global update strategy preserves estimation stability.

In Phase 2, the target vehicle executes a turning maneuver at the intersection, introducing stronger non-linearities and rapid yaw-rate changes. As a consequence, position errors increase across all methods due to model mismatch and the presence of correlated vehicle-level tracks. The Kalman Filter-based approaches (adapted EKF and adapted UKF) exhibit larger transient deviations in position and velocity estimation during this phase, reflecting their sensitivity to non-linear motion and implicit independence assumptions.

Phase 3 provides the most discriminative conditions for comparison. During this phase, one CAV produces degraded CPM estimates with significantly increased noise. The resulting heterogeneous input quality challenges the robustness of the fusion strategies. The ICI and IMF approaches are able to substantially reduce the influence of the degraded track, maintaining comparatively low position deviations. However, IMF exhibits increased velocity variability under degraded inputs.

In Phase 4, all fusion approaches converge toward similar position error levels, indicating stable behavior once the dynamic disturbances subside and input tracks regain consistency. These observations are reflected in the aggregated RMSE values summarized in Table 2, where ICI achieves the lowest overall position and velocity RMSE across the entire scenario.

**Figure 9:**
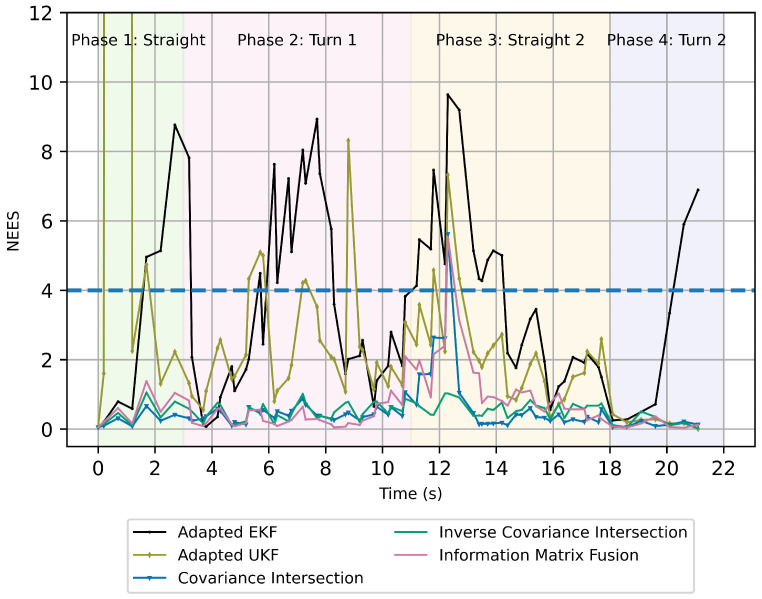
Normalized Estimation Error Squared (NEES) for track-to-track fusion algorithms using a peer-to-global fusion strategy. The dashed lines indicates the theoretical target NEES.

Figure 9 presents the Normalized Estimation Error Squared (NEES). NEES is a statistical consistency metric that evaluates whether the covariance reported by a state estimator correctly reflects the true estimation error. In track-to-track fusion, NEES is used to assess the consistency and reliability of fused tracks by comparing the estimation error against the predicted uncertainty, thereby indicating overconfidence or underconfidence in fused measurements. The theoretical target NEES is set to the length of the state vector (n=4), represented by a dashed line. Although the filter state vector is defined as ${\hat{x}}^{G}={\left[p_{x}^{G},p_{y}^{G},v^{G},\theta^{G},\psi^{G}\right]}^{T}$, the yaw-rate component $\psi^{G}$ is included only to support the CVTR motion model used in the prediction step. Since yaw rate is not directly observed in the measurement inputs, it is excluded from the consistency analysis. Consequently, the NEES analysis is performed only on the kinematic states $p_{x}^{G},p_{y}^{G},v^{G}$, and $\theta^{G}$, resulting in the target (n=4). The adapted EKF and UKF exhibit repeated overconfident covariance estimates, with the adapted UKF NEES (≈500) exceeding the plot upper limit in Phase 1. CI maintains low NEES values for most of the scenario indicating strongly conservative covariance estimates. ICI demonstrates the most stable NEES behavior among all methods. However, these values are below the expected NEES, indicating conservative uncertainty. IMF also maintains predominantly low NEES values below 2, with a moderate increase toward the theoretical bound during Phase 3.

## 6. Conclusions, Limitations, and Future Work

This work investigates track-to-track fusion strategies for cooperative perception using CPMs in a multi-CAV urban environment with non-linear vehicle motion and degraded-input CPMs. The presented methods were implemented in a peer-to-global fusion strategy as ROS2 nodes in a multi-phase scenario comprising a varying number of contributing CAVs, input accuracy, and target vehicle maneuvers.

The case study demonstrates behavioral differences between Kalman-based and intersection-based fusion approaches. The adapted EKF exhibits repeated exceedances of the theoretical NEES bound during dynamic phases, indicating periods of covariance underestimation and statistical inconsistency. The adapted UKF shows improved stability but still presents transient inconsistency during non-linear turning.

In contrast, CI, ICI, and IMF maintain relatively conservative NEES values throughout the scenario. These approaches avoid large inconsistency spikes, particularly during degraded input in Phase 3. However, their NEES values remain below the theoretical expectation, reflecting conservative uncertainty representations.

Regarding accuracy, all fusion methods substantially reduce the error compared to the degraded individual input. The RMSE values summarized in Table 2 indicate that ICI achieves the lowest overall position RMSE in case study, while CI and IMF demonstrate comparable performance. During the degraded-input phase, CI exhibits the largest transient peak error among the fusion approaches, whereas ICI and IMF limit this increase more effectively.

Overall, the results highlight the trade-off between statistical consistency and conservatism in decentralized fusion under unknown cross-correlations. While Kalman-based approaches may achieve competitive accuracy under nominal conditions, they are more sensitive to non-linear motion and correlated information. Intersection-based methods provide improved robustness at the expense of conservative covariance estimates. Table 2 summarizes the RMSE of the presented track-to-track fusion algorithms in the presented case study.

While the simulation-based case study presented in this work demonstrates the feasibility and effectiveness of peer-to-global high-level fusion strategies for cooperative perception using CPMs, several considerations remain for validating and extending the results to real-world scenarios. A key limitation lies in the idealized nature of the experimental setup. The CARLA simulator and Autoware framework used in our experiments approximate real-world conditions but do not replicate them entirely. This is especially true for V2X communication aspects such as latency, jitter, and bandwidth constraints. Despite this, an analysis of sensing and communication delays until CPM receipt at the fusion node with our CARLA–Autoware experiment setup indicated a latency of 280 ms. These results are consistent with real-world studies of CPM transmission delays between 226 and 426 ms [53]. Furthermore, the realism of perception remains constrained by limitations in the fidelity of simulated sensor raw data. While the focus of this study was not on simulating perception errors, this aspect is acknowledged as a source of divergence between simulation and real-world performance. Consequently, perception-related inaccuracies and their impact on fusion outcomes will require further investigation under more realistic conditions. Furthermore, results may vary under varying traffic density, road scenarios, CAV software and sensor configurations, and number of contributing ITS-S. As the number of participating ITS-S increases, the frequency of asynchronous updates to the global track may increase significantly. Although the algorithms evaluated here are computationally lightweight for the considered scenario, large-scale deployments will require further investigation of real-time scalability.

Looking ahead, future work will address these gaps through two key directions: First, future studies will include experiments with real-world CPMs to examine how the fusion modules respond to asynchronous inputs under variable conditions, communication delays, and sensor inconsistencies. Second, beyond the state fusion module analyzed in this study, we plan to implement the existence fusion and classification fusion modules that are key components of the high-level fusion architecture, which will enable more comprehensive representation of tracked objects and its associated uncertainty.

## Figures and Tables

**Table 1:** Characteristics of track-to-track fusion approaches for peer-to-global cooperative perception.

Fusion Method	Key Strengths	Key Limitations
CI	Guarantees estimator consistency when cross-correlation between tracks is unknown, making it suitable for decentralized and asynchronous inputs [16,36,37].	Produces conservative covariance estimates due to convex information weighting, which can limit accuracy improvements when high-quality and partially consistent measurements are available [36].
ICI	Provides less conservative covariance bounds than CI while maintaining estimator consistency, leading to improved robustness and accuracy in the presence of shared process noise [42,43].	Requires optimization of the fusion weight and assumes reliable covariance information from vehicle-level tracks, increasing computational complexity compared to CI [42].
Adapted KF	Computationally efficient and well suited for mildly non-linear motion models updated over small discrete time steps when asynchronous updates are handled through retrodiction [23,44,48].	Sensitive to non-linear maneuvers over large discrete time steps and correlated track inputs [39,45].
IMF	Explicitly accounts for information gain by retaining memory of previous updates, enabling robust fusion under noisy and partially inconsistent inputs [39,49].	Requires maintaining historical track information to compute information increments, increasing memory usage and implementation complexity [39].

**Table 2:** Average Root-Mean-Square Errors of position and velocity estimated from peer-to-global track-to-track fusion algorithms.

Algorithm	Position Average RMSE (m)	Velocity RMSE (m/s)
CI	0.616	0.53
ICI	0.17	0.419
Adapted EKF	0.316	0.56
Adapted UKF	0.366	0.49
Information Matrix Fusion	0.239	0.617

## Data Availability

Data sharing is not applicable.

## Abbreviations

The following abbreviations are used in this manuscript:

CAV	Connected and Automated Vehicle
CPM	Collective Perception Message
ITS-S	Intelligent Transport System Station
CVTR	Constant Velocity and Turn Rate
DST	Dempster Shafer Theory
ADAS	Advanced Driver Assistance System
CI	Covariance Intersection
ICI	Inverse Covariance Intersection
KF	Kalman Filter
EKF	Extended Kalman Filter
UKF	Unscented Kalman Filter
IMF	Information Matrix Fusion
